# Efficacy and Safety of PD-1 Inhibitor-Based Regimens in Patients with Melanoma: A Systematic Review and Meta-Analysis of Randomized Controlled Trials

**DOI:** 10.3390/jcm15124721

**Published:** 2026-06-17

**Authors:** Nikolaos Iasonas Kouris, Charalampos Filippatos, Konstantinos Lallas, Sofia Spyropoulou, Panagiotis Malandrakis, Evangelos Terpos, Maria Gavriatopoulou, Ioannis Ntanasis-Stathopoulos

**Affiliations:** 1 Department of Clinical Therapeutics, School of Medicine, National and Kapodistrian University of Athens, 11528 Athens, Greece; iaskouris@gmail.com (N.I.K.); panosmalandrakis@gmail.com (P.M.); eterpos@med.uoa.gr (E.T.);; 2 Department of Medical Oncology, Faculty of Health Science, School of Medicine, Aristotle University of Thessaloniki, 54124 Thessaloniki, Greece; koplallas@gmail.com; 3 Department of Dermatology, University Hospital of Tuebingen, 72076 Tuebingen, Germany; 4 Oncology Unit, 2nd Department of Surgery, Aretaieio University Hospital, School of Medicine, National and Kapodistrian University of Athens, 11528 Athens, Greece; sspyropoulou96@gmail.com

**Keywords:** melanoma, PD-1 inhibitors, immune checkpoint inhibitors, pembrolizumab, nivolumab, PD-L1

## Abstract

**Background:** Programmed death-1 (PD-1) inhibitors have significantly improved survival outcomes in melanoma; however, questions persist regarding comparative efficacy across regimens and the predictive value of PD-L1 expression as a biomarker. We therefore performed a meta-analysis evaluating outcomes according to PD-L1 expression status using the most recent follow-up data from each study. **Methods:** A systematic search was conducted in PubMed, Cochrane and ClinicalTrials.gov from 1 January 2010 to 1 April 2025 for phase II and III randomized clinical trials (RCTs) investigating PD-1 inhibitors as monotherapy or combined with other immune checkpoint inhibitors (ICIs) or targeted therapy in the adjuvant/metastatic setting. Pooled estimates were calculated with random-effects models, and risk of bias was assessed using the Cochrane RoB 2 tool. The present meta-analysis was performed following PRISMA guidelines and was registered in PROSPERO (ID: CRD420251090090). **Results:** Fifteen RCTs including 9979 patients were included. In the overall analysis, PD-1 inhibitors were associated with significantly improved overall survival (OS, HR = 0.75, 95% CI: 0.66–0.86) compared with control treatments. In the unresectable or metastatic setting, progression-free survival (PFS) was also significantly improved (HR = 0.61, 95% CI: 0.49–0.76). Survival benefits were observed in both PD-L1-positive and PD-L1-negative tumors, with improved PFS in PD-L1-positive (HR = 0.63, 95% CI: 0.48–0.83) and PD-L1-negative patients (HR = 0.58, 95% CI: 0.44–0.77), as well as improved OS in PD-L1-positive (HR = 0.69, 95% CI: 0.59–0.80) and PD-L1-negative patients (HR = 0.79, 95% CI: 0.67–0.93), without evidence of effect modification by PD-L1 expression. PD-1 inhibitor-based regimens were not associated with a statistically significant increase in grade 3–4 treatment-related adverse events (RR = 1.13, 95% CI: 0.71–1.79); however, heterogeneity was substantial (I^2^ = 96.0%). **Conclusions:** PD-1 inhibitor-based therapies significantly improve survival outcomes in advanced melanoma across PD-L1 subgroups. No clear evidence of differential treatment benefit according to PD-L1 expression was observed, suggesting limited utility as a standalone predictive biomarker. Further studies integrating molecular and immune profiling are warranted to optimize individualized treatment selection.

## 1. Introduction

The advent of immune checkpoint inhibitors (ICIs) targeting the programmed death-1 (PD-1) receptor has transformed the treatment of melanoma [1]. Agents such as nivolumab and pembrolizumab have demonstrated durable responses and improved survival in randomized controlled trials (RCTs), establishing PD-1 inhibitors as a standard of care for advanced melanoma [2,3].

Despite these advances, the use of PD-1 inhibitors in clinical practice-whether as monotherapy or in combination with other agents, such as CTLA-4 inhibitors-presents important challenges. While these regimens have been associated with improved efficacy, they are also accompanied by an increased incidence of immune-related adverse events (irAEs), such as elevated liver enzymes, pneumonitis, diarrhea and colitis, which can limit treatment tolerance and adversely affect patient outcomes [4,5]. This balance between efficacy and safety emphasizes the need to identify patient subgroups most likely to benefit from specific regimens, thereby improving patient outcomes while minimizing toxicity.

Among potential predictive biomarkers, programmed death-ligand 1 (PD-L1) expression has been extensively studied. However, its clinical utility as a predictive biomarker in melanoma remains uncertain, due to inconsistent findings across trials and heterogeneity in assay methods and cutoff thresholds [6].

In this context, we conducted a systematic review and meta-analysis of randomized clinical trials (RCTs) evaluating the efficacy and safety of PD-1 inhibitors in advanced or metastatic melanoma and in the adjuvant setting. In addition to synthesizing overall outcomes, we performed subgroup analyses, including stratification by PD-L1 expression status, using the most recent data available from each included study.

## 2. Materials and Methods

### 2.1. Protocol and Registration

This systematic review and meta-analysis was conducted in accordance with the Preferred Reporting Items for Systematic Reviews and Meta-Analyses (PRISMA) guidelines [7]. The study protocol was prospectively registered with the International Prospective Register of Systematic Reviews (PROSPERO; registration number: CRD420251090090). The completed PRISMA Checklist is provided in the Supplementary Materials (Supplementary Table S1).

### 2.2. Search Strategy

A systematic search was conducted in the databases PubMed, Cochrane Central Register of Controlled Trials and ClinicalTrials.gov, from 1 January 2010, given that the first PD-1 inhibitors received FDA approval in 2014 [8], to 1 April 2025, to identify full-text articles of RCTs on PD-1 inhibitor-based regimens for melanoma.

The search strategy used the following algorithm: (PD-1 inhibitors OR anti-PD-1 OR pembrolizumab OR nivolumab OR keytruda OR opdivo) AND melanoma. Records were screened independently by two reviewers at the title/abstract and full-text stages. Disagreements were resolved through team consensus. Searches were restricted to randomized controlled trials conducted in adult human populations and published in English.

Eligible articles included RCTs enrolling adult patients with stage III or IV melanoma in the adjuvant or metastatic setting, including first-line and previously treated populations, treated with PD-1 inhibitors either as monotherapy or combination with ICIs (including anti-CTLA-4 and anti-LAG3) or targeted therapy, and reporting efficacy and safety outcomes. Exclusion criteria comprised phase I trials, studies focusing exclusively on non-cutaneous melanoma, studies conducted in the neoadjuvant setting, studies including stage IIB/IIC stage only, dosing studies, case reports, reviews, in vitro or animal studies, and records not available in English. Terminated trials were also excluded due to incomplete outcome data.

### 2.3. Data Extraction

Data extraction included the following: study characteristics (first author, publication year, study ID, geographic region, sample size, follow-up duration), population characteristics (number of males, age), interventions and comparators, efficacy and safety outcomes [overall survival (OS), progression-free survival (PFS), recurrence-free survival (RFS) and grade 3 or 4 treatment-related adverse events (TRAEs)] for the overall study population and PD-L1 subgroups. Hazard ratios (HRs), risk ratios (RRs), and corresponding 95% confidence intervals (CIs) were collected. If RRs were not available, crude numbers were extracted and the ratios were subsequently calculated within the meta-analysis. For each study, the most recent follow-up data were included.

In case the data were not available in the main text, the Supplementary Material was thoroughly reviewed to retrieve the corresponding results. Data were independently extracted, analyzed and recorded by two reviewers. The finalized data form was reached after team consensus.

### 2.4. Statistical Analysis

Statistical analyses included the pooling of studies, subsequent subgroup analyses as well as post hoc meta-regressions. Random-effects models were used to calculate the pooled effect estimates (HRs and RRs). Between-study heterogeneity was assessed by Q-test and I^2^ estimations. Pre-specified subgroup analyses were conducted based on trial phase, dosing, adjustment, treatment regimen, treatment setting and PD-L1 tumor expression status. Furthermore, to account for methodological variability in biomarker ascertainment, an additional subgroup analysis was performed stratifying the studies by the specific PD-L1 expression cutoff thresholds used to define positivity in each trial. Post hoc meta-regression analyses were performed for subgroups with a total of 10 or more data entries for the variables to be analyzed, including age, follow-up period, percentage of males and number of patients.

The primary outcomes were PFS and OS for metastatic melanoma, RFS and OS for the adjuvant setting and AEs. Comparisons were conducted between PD-1 inhibitor-based regimens, administered either as monotherapy or in combination with other ICIs or targeted therapy, and control treatments, including other ICIs, chemotherapy, targeted therapy, or placebos.

In multi-arm trials where more than one experimental regimen was compared with a single, shared control group, adjustments were made to prevent double-counting of the control population. The experimental arms within these specific studies were combined prior to the main analysis. A within-study fixed-effect meta-analysis was performed on the experimental groups to generate a single composite effect size and variance (hazard ratios for survival outcomes and risk ratios for safety outcomes) for each trial.

All statistical analyses were performed using R/R-Studio version 2024.04.2+764 (Posit Software, PBC, Boston, MA, USA).

### 2.5. Risk of Bias

The risk of bias in individual studies was evaluated using the Cochrane Risk of Bias tool (RoB 2.0) [9]. Domains assessed included randomization process, deviations from intended interventions, missing outcome data, measurement of the outcome, and selection of the reported result. Judgments were categorized as “low”, “some concerns”, or “high” risk of bias. The certainty of evidence for each outcome was assessed using the GRADE approach [10].

## 3. Results

### 3.1. Study Selection

**Figure 1:**
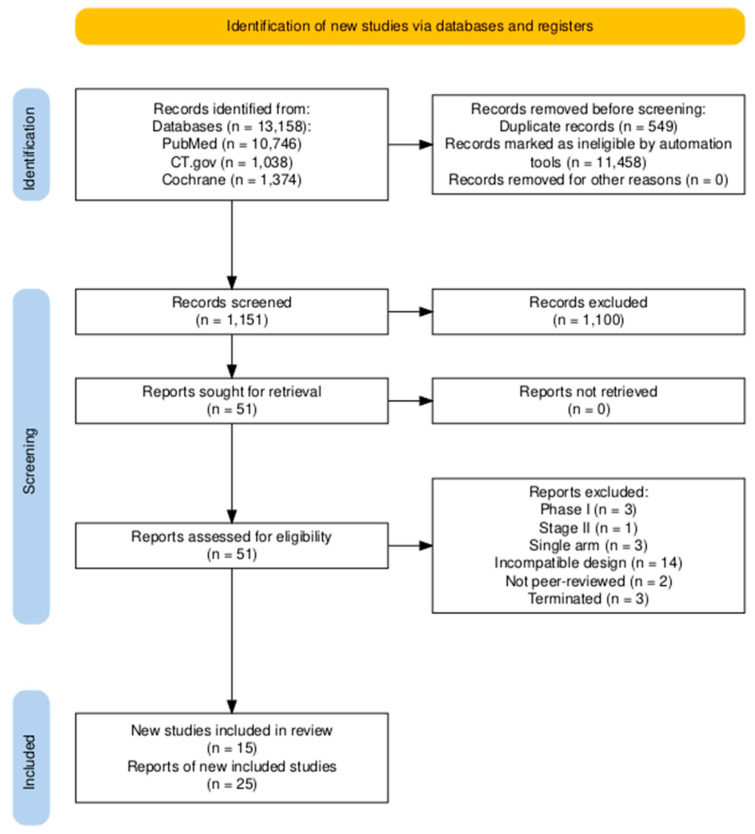
PRISMA 2020 Flow Diagram.

The initial database search identified a total of 13,158 records. After removal of duplicates and the application of automation tools, 1151 records were retained for initial screening. Of these, 1100 records were excluded, while 51 full-text articles were reviewed for eligibility. Ultimately, 25 publications from 15 RCTs met the full eligibility criteria and were included in the analysis [3,11,12,13,14,15,16,17,18,19,20,21,22,23,24,25,26,27,28,29,30,31]. The PRISMA Flow Diagram (Figure 1) outlines the study selection process.

### 3.2. Study Characteristics

The 15 selected RCTs included a total of 9979 patients. All studies included adults with histologically confirmed advanced or metastatic melanoma. Five studies [18,20,21,25,26] evaluated PD-1 inhibitors in the adjuvant setting, whereas the remaining studies enrolled patients with unresected or metastatic melanoma. Sample sizes ranged from 92 to 1844 participants, with a median follow-up duration of 13 to 61 months. Median patient age ranged from 54 to 67 years, with a slight male predominance (51–65%). Table 1 presents an overview of the included studies.

### 3.3. Overall Survival

In the overall random-effects meta-analysis, including both adjuvant and metastatic settings, ICI-based therapies were associated with a significant improvement in overall survival (OS) compared with control treatments (HR = 0.75, 95% CI: 0.66–0.86). Moderate heterogeneity was observed (I^2^ = 67.4%, *p* = 0.0001). Meta-regression of the overall treatment effect showed that higher study-level median age was associated with a greater overall survival benefit (β = −0.0388, *p* = 0.022) (Supplementary Table S2).

#### 3.3.1. Subgroup Analyses by Treatment Regimen

**Figure 2:**
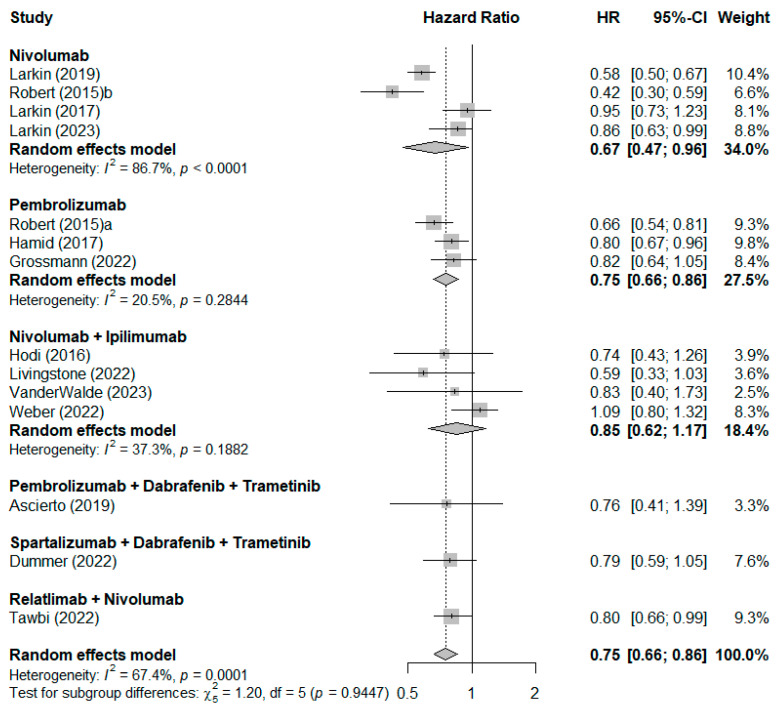
Forest plot of hazard ratios for overall survival by treatment strategy.

In subgroup analyses, both pembrolizumab monotherapy (HR = 0.75, 95% CI: 0.66–0.86) and nivolumab monotherapy (HR = 0.67, 95% CI: 0.47–0.96) demonstrated statistically significant OS benefits compared with ipilimumab or chemotherapy. Nivolumab plus ipilimumab showed trends toward improved OS, although the analysis did not reach statistical significance (HR = 0.85, 95% CI: 0.62–1.17) (Figure 2). Combination regimens involving BRAF/MEK inhibitors and ICIs did not demonstrate statistically significant survival benefits. The test for subgroup differences found no evidence of variation in treatment effect among the therapeutic subgroups (*p* = 0.9447). In addition, subgroup analyses revealed a statistically significant difference in overall survival according to the dosing schedule (*p* < 0.0001), with the combined Q2W/Q3W regimen demonstrating the greatest benefit (HR = 0.61, 95% CI: 0.54–0.68) (Supplementary Table S4).

#### 3.3.2. Subgroup Analyses by PD-L1 Status

**Figure 3:**
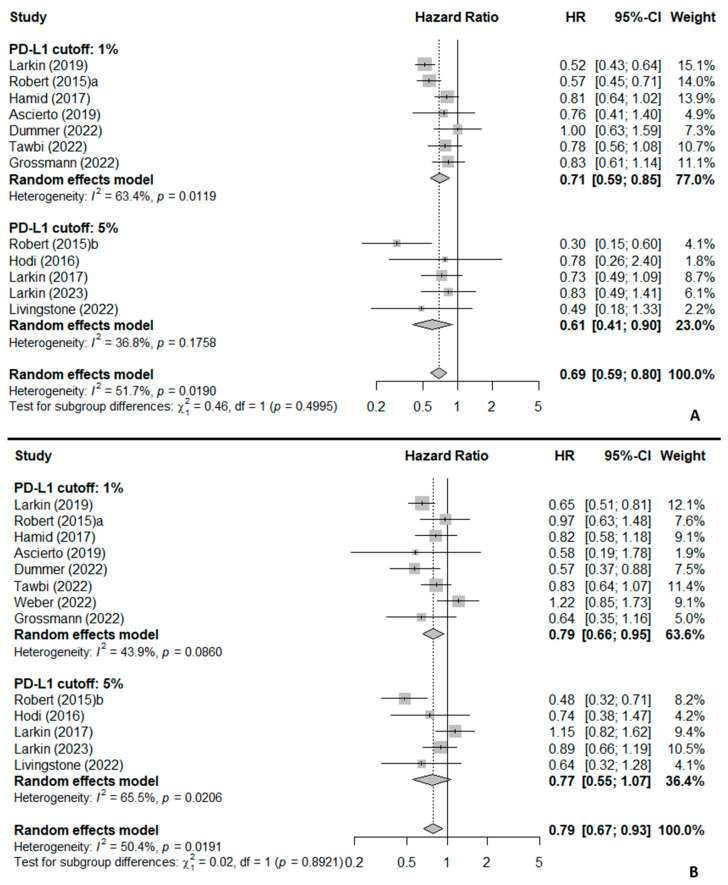
Subgroup analysis of overall survival among PD-L1 (**A**) positive and (**B**) negative patients.

In subgroup analyses stratified by PD-L1 expression, PD-1 inhibitor-based therapies were associated with improved OS in both PD-L1-positive and PD-L1-negative tumors. Among patients with PD-L1-positive tumors, treatment with PD-1 inhibitors was associated with a significant reduction in mortality (HR = 0.69, 95% CI: 0.59–0.80), with moderate heterogeneity (I^2^ = 51.7%). No significant differences in treatment effects across PD-L1 cutoff thresholds was observed in this subgroup (*p* = 0.500) (Figure 3A). Furthermore, among patients with PD-L1-positive tumors, overall survival benefit varied significantly according to the dosing schedule (*p* = 0.001) (Supplementary Table S7). In patients with PD-L1-negative tumors, PD-1 inhibitor-based regimens were also associated with improved OS (HR = 0.79, 95% CI: 0.67–0.93), with low to moderate heterogeneity (I^2^ = 50.4%). No statistically significant differences in treatment effects were observed across PD-L1 cutoff thresholds (*p* = 0.892) (Figure 3B).

Comparison of pooled hazard ratios between PD-L1-positive and PD-L1-negative subgroups did not demonstrate a statistically significant difference in treatment effect for OS (HR difference = 0.10; *p* = 0.236), indicating no evidence of effect modification by PD-L1 expression.

#### 3.3.3. Subgroup Analyses by Treatment Setting

**Figure 4:**
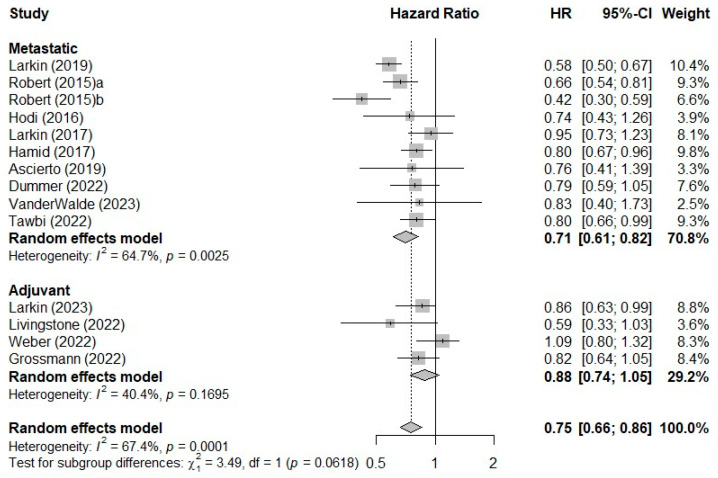
Forest plot of hazard ratios for overall survival by treatment setting.

In subgroup analyses stratified by treatment setting, PD-1 inhibitors were associated with a significant improvement in OS in the metastatic setting (HR = 0.71, 95% CI: 0.61–0.82), while the effect in the adjuvant setting was not statistically significant (HR = 0.88, 95% CI: 0.74–1.05). (Figure 4) The test for subgroup differences did not reach statistical significance (*p* = 0.0618).

Further subgroup analyses were conducted according to PD-L1 expression within each treatment setting. In both PD-L1-positive and PD-L1-negative patients, a significant survival benefit was observed in the metastatic setting (PD-L1-positive: HR = 0.67, 95% CI: 0.55–0.80; PD-L1-negative: HR = 0.73, 95% CI: 0.61–0.87), whereas no statistically significant benefit was demonstrated in the adjuvant setting (PD-L1-positive: HR = 0.80, 95% CI: 0.62–1.04; PD-L1-negative: HR = 0.89, 95% CI: 0.67–1.19). No significant differences according to treatment setting were detected in either subgroup.

### 3.4. Progression-Free Survival

In the overall random-effects meta-analysis, PD-1 inhibitor-based regimens were associated with a significant improvement in progression-free survival (PFS) compared with control treatments (HR = 0.61, 95% CI: 0.49–0.76). Substantial heterogeneity was observed across studies (I^2^ = 83.4%). As PFS data were available only in unresected/metastatic studies, analyses were limited to this population.

#### 3.4.1. Subgroup Analyses by Treatment Regimen

**Figure 5:**
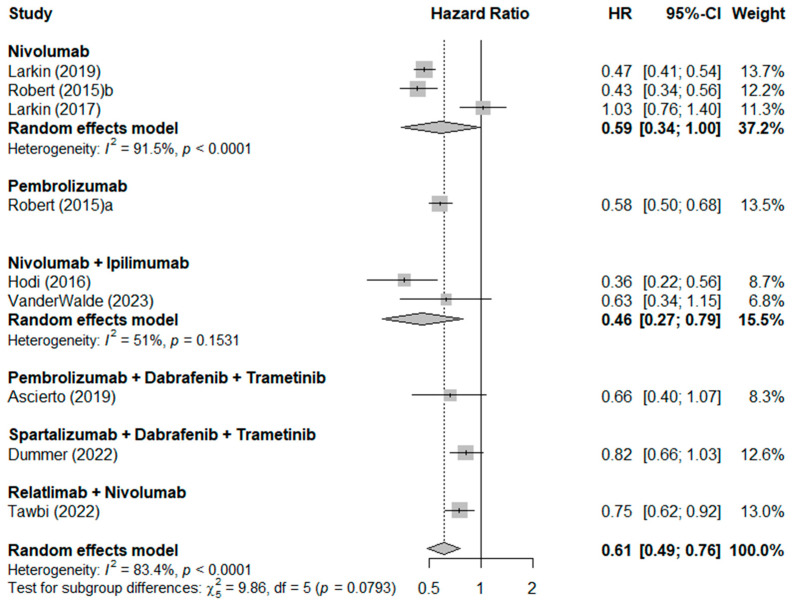
Forest plot of hazard ratios for progression-free survival by treatment strategy.

In regimen-specific analyses, nivolumab plus ipilimumab demonstrated a significant improvement in PFS (HR = 0.46, 95% CI: 0.27–0.79). Pembrolizumab monotherapy was also associated with a statistically significant PFS benefit (HR = 0.58, 95% CI: 0.50–0.68), whereas nivolumab monotherapy showed a borderline statistically significant improvement in PFS (HR = 0.59, 95% CI: 0.34–1.00). Combination regimens incorporating BRAF and MEK inhibitors did not demonstrate statistically significant improvements in PFS. (Figure 5) No statistically significant difference in treatment effects across therapeutic subgroups was observed (*p* = 0.0793).

#### 3.4.2. Subgroup Analyses by PD-L1 Status

**Figure 6:**
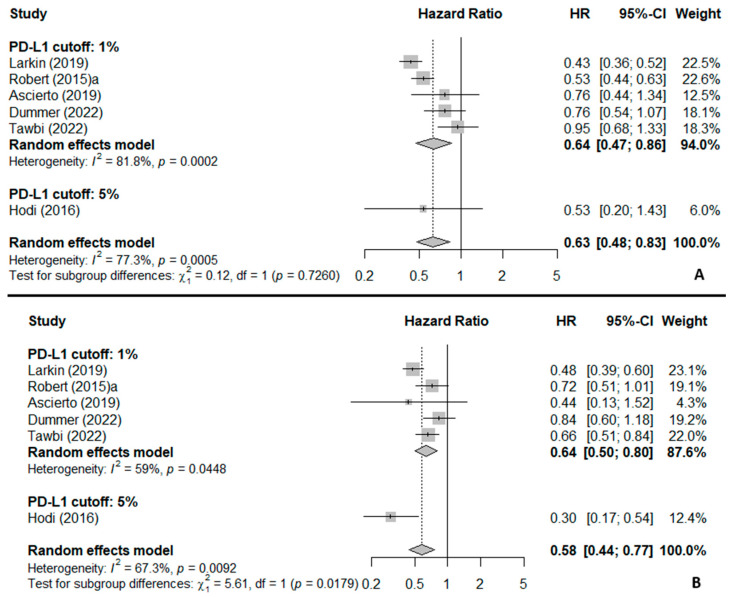
Subgroup analysis of progression-free survival among PD-L1 (**A**) positive and (**B**) negative patients.

In subgroup analyses stratified by PD-L1 expression status, PD-1 inhibitor-based therapies were associated with significant improvements in PFS in both PD-L1-positive and PD-L1-negative tumors. Among patients with PD-L1-positive tumors, treatment was associated with a significant improvement in PFS (HR = 0.63, 95% CI: 0.48–0.83), with substantial heterogeneity (I^2^ = 77.3%). No significant differences in treatment effects were observed across PD-L1 cutoffs (*p* = 0.726) (Figure 6A). Similar to overall survival, progression-free survival benefit in this subgroup varied significantly according to the dosing schedule (*p* = 0.001) (Supplementary Table S9). In patients with PD-L1-negative tumors, PD-1 inhibitors were also associated with improved PFS (HR = 0.58, 95% CI: 0.44–0.77), with moderate to high heterogeneity (I^2^ = 67.3%). Subgroup analysis demonstrated significant differences in treatment effects across PD-L1 cutoffs (*p* = 0.018) (Figure 6B). Additionally, a significant difference in progression-free survival benefit was observed across trial phases for PD-L1-negative patients (*p* = 0.018), with phase II trials reporting a more pronounced effect (HR = 0.32, 95% CI: 0.19–0.54) compared to phase III trials (HR = 0.65, 95% CI: 0.51–0.82) (Supplementary Table S10). No difference in treatment effect on PFS was observed between PD-L1-positive and PD-L1-negative subgroups (HR difference = −0.05; *p* = 0.598), suggesting that PD-L1 expression does not modify PFS benefit.

### 3.5. Recurrence-Free Survival

Five adjuvant trials reported recurrence-free survival (RFS) [18,20,21,25,26]. The pooled hazard ratio demonstrated a significant reduction in the risk of recurrence with PD-1 therapy (HR = 0.69, 95% CI: 0.54–0.86), although substantial heterogeneity was observed (I^2^ = 83.7%, *p* < 0.0001).

In subgroup analyses according to PD-L1 expression, the HR was 0.68 (95% CI: 0.55–0.84) in PD-L1-positive patients and 0.70 (95% CI: 0.51–0.95) in PD-L1-negative patients. No significant difference in treatment effect on RFS was observed between PD-L1-positive and PD-L1-negative subgroups.

The corresponding forest plots are presented in Supplementary Figures S1 and S2.

### 3.6. Safety

**Figure 7:**
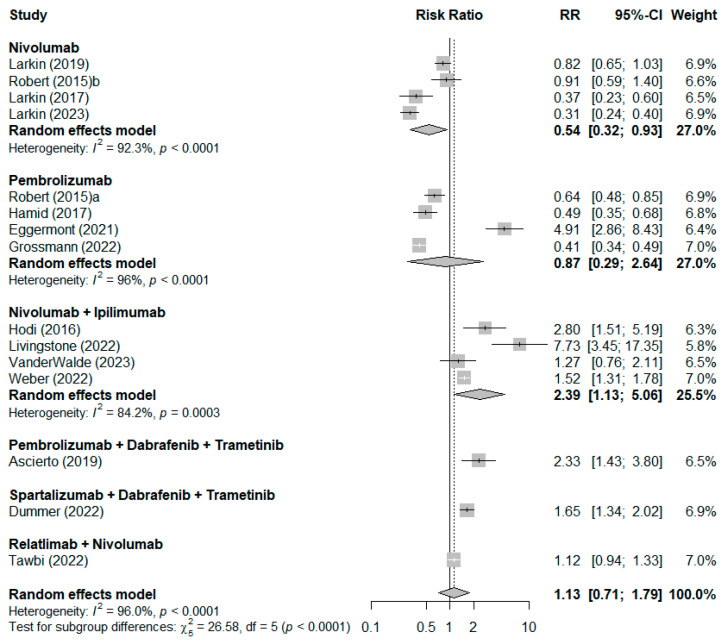
Forest plot of risk ratios for grade 3 or 4 adverse events by treatment strategy.

In the overall random-effects meta-analysis, PD-1 inhibitor-based regimens were not associated with a statistically significant increase in the risk of grade 3 or 4 adverse events compared with control treatments (RR = 1.13, 95% CI: 0.71–1.79). However, considerable heterogeneity was observed across studies (I^2^ = 96.0%) (Figure 7). A statistically significant difference in adverse event risk was observed across treatment subgroups (*p* < 0.0001).

In subgroup analyses by treatment regimen, nivolumab monotherapy was associated with lower risk of grade 3–4 AEs when compared with ipilimumab, chemotherapy or placebos (RR = 0.54, 95% CI: 0.32–0.93). Pembrolizumab monotherapy was not associated with a statistically significant difference in AEs (RR = 0.87, 95% CI: 0.29–2.64).

Nivolumab plus ipilimumab showed a significantly increased risk of grade 3–4 AEs when compared with ipilimumab or nivolumab monotherapy (RR = 2.39, 95% CI: 1.13–5.06).

Regimens combining ICIs with BRAF/MEK inhibitors were associated with higher incidence of AEs, although these results were derived from single studies and should be interpreted cautiously.

In subgroup analyses according to dosing schedule, the Q2W regimen was associated with a significantly reduced risk of grade 3–4 adverse events (RR = 0.47, 95% CI: 0.24–0.90). The test for subgroup differences was statistically significant (*p* = 0.003), suggesting that the risk of grade 3–4 adverse events vary according to dosing schedule (Supplementary Table S6).

Meta-regression analyses for grade 3 or 4 AEs showed no statistically significant associations between the examined study-level variables (age, follow-up period, percentage of males and number of patients) and treatment effect, suggesting no evidence of effect modification (Supplementary Table S3).

### 3.7. Risk of Bias Assessment

All RCTs were assessed using the RoB:2 tool and demonstrated an overall low risk of bias across most domains, particularly in the randomization process, outcome measurement, and selection of the reported results. Some concerns were noted specifically in the domain of deviations from intended interventions, related to open-label design and high rates of crossover. For example, in CheckMate 037, higher rates of crossover and withdrawal of consent before treatment initiation were observed in the chemotherapy group, potentially diluting treatment effects. No study was judged to be at high overall risk of bias. A detailed traffic light plot is provided in Supplementary Table S12.

The certainty of evidence was assessed using the GRADE approach and is presented in Supplementary Table S13.

## 4. Discussion

This systematic review and meta-analysis demonstrates that PD-1 inhibitor-based therapies are associated with a 25% lower risk of death and a 39% reduction in the risk of disease progression or death compared with other ICIs, targeted therapy, chemotherapy or placebos. These findings further support the established role of PD-1 inhibitors as standard therapy in advanced melanoma.

A primary objective of this analysis was to evaluate PD-L1 expression as a predictive biomarker. PD-L1 subgroup analyses included 14 studies comprising 5858 PD-L1-positive patients and 3499 PD-L1-negative patients. Statistically significant OS and PFS benefits were observed in both PD-L1-positive and PD-L1-negative tumors. However, direct comparisons between these subgroups did not demonstrate statistically significant effect modification. Therefore, no clear evidence of differential treatment benefit according to PD-L1 expression status was observed in the present analysis. These findings support current guideline recommendations that PD-L1 testing is not required prior to initiation of PD-1 inhibitor therapy in melanoma [32]. However, these findings should be interpreted cautiously, given the variability in PD-L1 assessment methods across studies. Moreover, in subgroup analyses stratified by PD-L1 expression-status, the magnitude of benefit varied across treatment regimens. In PD-L1-positive patients, statistically significant improvements in PFS were observed with nivolumab and pembrolizumab, whereas in PD-L1 negative patients, significant benefits were observed with nivolumab, nivolumab plus ipilimumab, and relatlimab plus nivolumab. However, no significant effect modification according to PD-L1 expression was observed.

Specifically, in RELATIVITY-047, relatlimab (a LAG-3 inhibitor) plus nivolumab demonstrated a significant improvement in both OS and PFS in the metastatic setting compared with nivolumab monotherapy [24]. Although greater PFS benefit was observed in PD-L1-negative patients, this pattern was not observed for OS. These findings also support the exploration of combination ICIs in PD-L1 negative tumors in future studies. In the more recent RELATIVITY-098, which was not included in the present meta-analysis, nivolumab plus relatlimab did not significantly improve RFS over nivolumab as adjuvant treatment for patients after complete resection of stage III-IV melanoma. No differences in RFS were observed across PD-L1 expression subgroups [33].

The overall analysis did not demonstrate any statistically significant increase in grade 3–4 adverse events with PD-1-based therapies compared with control treatments; however, heterogeneity was very high. In addition, several studies included active immunotherapy comparators, such as nivolumab or ipilimumab, which may have attenuated the observed differences in toxicity between groups. Thus, the absence of a statistically significant increase in grade 3–4 adverse events should be interpreted with caution. Although nivolumab monotherapy was associated with a lower incidence of grade 3–4 adverse events, heterogeneity was substantial (I^2^ = 92.3%), likely reflecting differences in comparator treatments. A greater safety advantage would be expected when nivolumab is compared with ipilimumab or chemotherapy than with the placebo. A lower risk of grade 3–4 adverse events was also observed with Q2W dosing; however, given the substantial heterogeneity and differences in treatment regimens (nivolumab and pembrolizumab) and control regimens (ipilimumab and chemotherapy) across studies, this finding should be interpreted cautiously. Sub-group analyses also suggested greater toxicity with nivolumab plus ipilimumab compared with PD-1 monotherapy. This finding is broadly consistent with existing literature, demonstrating higher toxicity with combination therapy compared with PD-1 monotherapy [34].

Several trials are exploring methods of improving the tolerability of combination therapy, such as using low-dose ipilimumab [35]. The CheckMate 511 trial evaluated an alternative “flipped” dosing regimen (nivolumab 3 mg/kg plus ipilimumab 1 mg/kg) and demonstrated a significantly improved safety profile compared with the standard regimen (nivolumab 1 mg/kg plus ipilimumab 3 mg/kg), while maintaining comparable efficacy [36].

Moreover, in the subgroup analysis by treatment setting, a statistically significant overall survival benefit was observed in the metastatic setting, whereas the effect in the adjuvant setting did not reach statistical significance. This may reflect lower event rates and longer follow-up requirements in patients with resected tumors, with recurrence-free survival likely serving as a more sensitive endpoint in the adjuvant setting. Although an overall pooled OS effect was estimated, the adjuvant and metastatic settings should be interpreted separately in clinical practice, given the substantial differences in patient characteristics, endpoints, and event rates between these populations. Furthermore, treatment effects varied according to therapeutic strategy, with nivolumab plus ipilimumab demonstrating the greatest progression-free survival benefit, while pembrolizumab monotherapy showed consistent improvements in both OS and PFS. Comparator treatments varied substantially across studies, including the placebo, chemotherapy, targeted therapy, and immunotherapy. This analysis did not evaluate distant metastasis-free survival (DMFS), which may provide additional clinically meaningful insight in the absence of an OS benefit.

The observed association between higher study-level median age and greater overall survival benefit should be interpreted with caution. Although this finding may be consistent with emerging biological and clinical evidence suggesting that age-related changes in the tumor immune microenvironment—such as reduced immunosuppressive regulatory T-cell activity—could potentially enhance responsiveness to PD-1 blockade [37], this explanation remains speculative.

The present work synthesizes the most recent and complete data on survival outcomes stratified by PD-L1 expression status, aiming to inform contemporary clinical practice regarding the role of PD-L1 as a biomarker and guide therapeutic decisions using the latest follow-up data available from each trial. This meta-analysis included fifteen trials, encompassing 9979 patients in total, thereby enhancing the statistical power of the pooled estimates. Only phase II and III RCTs were included, ensuring the methodological robustness of the analysis. An additional strength of this meta-analysis is that comprehensive subgroup and meta-regression analyses were conducted to explore potential sources of heterogeneity, which appears to be significant in PD-1 inhibitor trials, and effect modification.

Several limitations should be acknowledged, as well. Substantial heterogeneity was observed in multiple analyses, particularly for PFS and adverse events, likely reflecting differences in study patient populations, treatment lines, comparator regimens, crossover rates, follow-up duration and adverse event reporting. Such heterogeneity may limit the generalizability and clinical interpretation of the pooled estimates. Moreover, neoadjuvant trials were excluded from this analysis because they differ substantially in treatment setting and outcome measurement from adjuvant and unresectable trials, which would introduce additional heterogeneity. In addition, PD-L1 subgroup analyses were limited by variability in the definition of PD-L1 positivity across trials. Included studies used different cutoff thresholds (e.g., ≥1% or ≥5%). For the purposes of this meta-analysis, PD-L1-positive and PD-L1-negative subgroups were analyzed according to the definitions provided in each individual trial, potentially reducing the precision of biomarker-related estimates. To address this limitation, additional subgroup analyses were performed according to the PD-L1 positivity cutoff used in each study. Although no significant differences in treatment effects were observed between studies using 1% and 5% thresholds, adoption of more uniform PD-L1 assessment methods and cutoff thresholds across future trials would facilitate more reliable biomarker evaluation and improve comparability between studies. Finally, although the most recent 10-year follow-up publications of CheckMate 069 and KEYNOTE-006 provide important updated survival data, they were not included in this meta-analysis because PFS or OS outcomes were not reported according to PD-L1 subgroups [11,28]. As our study specifically focused on PD-L1 subgroup analyses, we instead chose to use earlier reports, which provided the required data. This approach also avoided combining results from different follow-up periods. Nevertheless, shorter follow-up periods may underestimate the survival benefit and limit the interpretation of OS.

In conclusion, this meta-analysis demonstrates that PD-1 inhibitor-based therapies significantly improve survival outcomes in advanced melanoma across PD-L1 subgroups. No clear evidence of differential treatment benefit according to PD-L1 expression was observed, suggesting limited utility as a standalone predictive biomarker. Further studies integrating molecular and immune profiling are warranted to optimize individualized treatment selection.

## Figures and Tables

**Table 1:** Characteristics of the included studies.

Study	Author (Year)	Phase	Drug	Control	*N*	Age	FUP
CheckMate 067	Larkin et al. (2019) [30]	III	Arm A: Nivolumab 3 mg/kg Q2W	Ipilimumab 3 mg/kg Q3W	945	60	36
			Arm B: Nivolumab 1 mg/kg Q3W + Ipilimumab 3 mg/kg Q3W				54.6
KEYNOTE 006	Robert et al. (2015) [12]	III	Arm A: Pembrolizumab 10 mg/kg Q2W	Ipilimumab 3 mg/kg Q3W	834	60	57.7
			Arm B: Pembrolizumab 10 mg/kg Q3W				
CheckMate 066	Robert et al. (2015) [13]	III	Nivolumab 3 mg/kg Q2W	Dacarbazine	418	65	32
CheckMate 069	Hodi et al. (2016) [14]	II	Nivolumab 1 mg/kg Q3W + Ipilimumab 3 mg/kg Q3W	Ipilimumab	142	64	24
CheckMate 037	Larkin et al. (2017) [16]	III	Nivolumab 3 mg/kg Q2W	Chemotherapy	405	59	24
KEYNOTE 002	Hamid et al. (2017) [31]	II	Arm A: Pembrolizumab 2 mg/kg Q3W	Chemotherapy	540	60	28
			Arm B: Pembrolizumab 10 mg/kg Q3W				
CheckMate 238	Larkin et al. (2023) [18]	III	Nivolumab 3 mg/kg Q2W	Ipilimumab 10 mg/kg Q3W	906	54	61.5
KEYNOTE 022	Ascierto et al. (2019) [19]	II	Pembrolizumab 2 mg/kg + DabTram	DabTram	120	56	36.6
IMMUNED	Livingstone et al. (2022) [20]	II	Arm A: Nivolumab 1 mg/kg Q3W + Ipilimumab 3 mg/kg Q3W	Placebo	167	55	28.4
			Arm B: Nivolumab 3 mg/kg Q2W				
EORTC 1325-MG/KEYNOTE 054	Eggermont et al. (2021) [21]	III	Pembrolizumab 200 mg Q3W	Placebo	1019	54	42.3
COMBI-i	Dummer et al. (2022) [22]	III	Spartalizumab 400 mg Q3W + DabTram	DabTram	532	55	27.2
SWOG S1616	VanderWalde et al. (2023) [23]	II	Nivolumab 1 mg/kg Q3W + Ipilimumab 3 mg/kg Q3W	Ipilimumab 3 mg/kg Q3W	92	67	28
RELATIVITY-047	Tawbi et al. (2022) [24]	III	Relatlimab 160 mg + Nivolumab 480 mg Q4W	Nivolumab 480 mg Q4W	714	61	13.2
CheckMate 915	Weber et al. (2022) [25]	III	Nivolumab 240 mg Q2W + Ipilimumab 1 mg/kg Q6W	Nivolumab 480 mg Q4W	1844	55	28.1
SWOG S1404	Grossmann et al. (2022) [26]	III	Pembrolizumab 200 mg Q3W	IFN-a or Ipilimumab 10 mg/kg Q3W	1301	57	47.5

## Data Availability

Data are available from the corresponding author upon reasonable request.

## Supplementary Materials

The following supporting information can be downloaded at https://www.mdpi.com/article/10.3390/jcm15124721/s1: Table S1. PRISMA 2020 Checklist; Table S2. Meta-regression analysis for overall survival (OS); Table S3. Meta-regression analysis for grade 3–4 adverse events (AEs); Table S4. Subgroup analysis of overall survival (OS); Table S5. Subgroup analysis of progression-free survival (PFS); Table S6. Subgroup analysis of grade 3–4 adverse events (AEs); Table S7. Subgroup analysis of OS in PD-L1-positive patients; Table S8. Subgroup analysis of OS in PD-L1-negative patients; Table S9. Subgroup analysis of PFS in PD-L1-positive patients; Table S10. Subgroup analysis of PFS in PD-L1-negative patients; Table S11. Test differences between PD-L1 negative vs. PD-L1 positive; Table S12. Risk of bias assessment (RoB:2); Table S13. Certainty of Evidence (GRADE); Figure S1. Forest plot of hazard ratios for recurrence-free survival (RFS); Figure S2. Sensitivity analysis of recurrence-free survival (RFS) according to PD-L1 expression status; Figure S3. Subgroup analysis of RFS by PD-L1 cutoff thresholds; Figure S4. Subgroup analysis of OS in PD-L1-positive patients by treatment regimen; Figure S5. Subgroup analysis of OS in PD-L1-negative patients by treatment regimen; Figure S6. Subgroup analysis of PFS in PD-L1-positive patients by treatment regimen; Figure S7. Subgroup analysis of PFS in PD-L1-negative patients by treatment regimen.
